# Improved application technique of albumin-glutaraldehyde glue for repair of superficial lung defects

**DOI:** 10.1186/s13019-016-0544-6

**Published:** 2016-10-21

**Authors:** Maximilian Bures, Patrick Zardo, Florian Länger, Ruoyu Zhang

**Affiliations:** 1 Department of Cardiac, Thoracic, Transplantation and Vascular Surgery, Hannover Medical School, Hannover, Germany; 2 Department of Cardiac and Thoracic Surgery, Otto-von-Guericke University Magdeburg, Magdeburg, Germany; 3 Department of Pathology, Hannover Medical School, Carl-Neuberg Str. 1, 30625 Hannover, Germany; 4 Department of Thoracic Surgery, Center for Pneumology and Thoracic Surgery, Chest Hospital Schillerhoehe, Teaching hospital of the University of Tuebingen, Gerlingen, Germany; 5 Department of Thoracic Surgery, Center for Pneumology and Thoracic Surgery, Schillerhoehe Hospital, Solitudestr. 18, Gerlingen, Germany

**Keywords:** Lung, Air leaks, Sealant, BioGlue, Application

## Abstract

**Background:**

Albumin-glutaraldehyde glue has gained widespread acceptance for treatment of alveolar air leaks (AAL) in thoracic surgery. As liquid run-off during application is detrimental to its sealing efficacy, we developed a modified technique and assessed it in vitro.

**Methods:**

Caudal lobes of freshly excised swine lungs (*n* = 20) were intubated and ventilated. A standardized focal superficial parenchymal defect (40 × 25 mm) was created on the inflated lung. AAL was assessed under exposure to increasing inspired tidal volume (TVi). Lung lobes were randomly selected and subjected to either a standard sealing suggested by the manufacturer (control group) or a modified technique relying on placement of a square silicone frame around the lesion site (study group). AAL was subsequently assessed until burst failure occurred and the occuring lesions length was recorded on the inflated lung to evaluate elasticity of underlying tissue.

**Results:**

Superficial parenchymal defects resulted in AAL increasing with ascending TVi. AAL prior to sealant application was comparable in both groups. An application error occurred once in our control group. At TVi = 400, 500, 600 and 700 ml, the albumin-glutaraldehyde glue achieved complete sealing in 10, 10, 9 and 8 lungs respectively in our study group, as opposed to 9, 7, 6 and 4 lobes in the control group. The required mean burst pressure was significantly higher in our study group (41.0 ± 1.0 vs. 37.5 ± 4.2 cmH_2_O, *p* = 0.0195), but there was no difference in expansion of covered defect between both groups (1.0 ± 0.4 vs. 1.5 ± 1.7 mm, *p* = 0.3772).

**Conclusions:**

Our tests suggest that frame-assisted sealant application might prevent glue run-off and thus improves its sealing efficacy. We encourage further investigation of this technique in well-designed, controlled clinical trials.

**Electronic supplementary material:**

The online version of this article (doi:10.1186/s13019-016-0544-6) contains supplementary material, which is available to authorized users.

## Abstract in German

**Hintergrund:**

Albumin-Glutaraldehyd-Kleber ist zunehmend bei Behandlung des alveolo-pleuralen Luftlecks (AAL) in der Lungenchirurgie eingesetzt. Häufig kommt bei der Applikation unkontrolliertes Abfließen dieses flüssigen Klebers vor und führt zu Beeinträchtigung der Abdichtungswirksamkeit. Wir evaluierten in dieser Studie eine rahmengestützte Applikationstechnik mittels in vitro Lungenmodels.

**Methode:**

Der kaudale Lappen der frisch entnommenen Schweinlungen (*n* = 20) wurde intubiert und beatmet. Eine pleurale Läsion (40 × 25 mm) wurde erstellt und AAL mit steigendem inspiratorischem Tidalvolumen (TVi) untersucht. Die Lungenlappen wurden randomisiert mit Standardverfahren (Kontrollgruppe) oder mit der rahmengestützten Technik appliziert (Versuchsgruppe), wobei ein quadratischer Silikonrahmen um die Läsion platziert wurde. Nach Applikation von Albumin-Glutaraldehyd-Kleber wurde AAL auf die gleiche Weise gemessen bis zur Auftritt von Kleberbruch. Zur Untersuchung der Elastizität des Klebers wurde die Länge der pleuralen Läsion gemessen.

**Ergebnis:**

Die oberflächliche pleurale Läsion führte bei aufsteigendem TVi zum Anstieg von AAL. Vor der Applikation des Klebers war AAL vergleichbar in den beiden Gruppen. Applikationsfehler trat bei einem Test in der Kontrolgruppe auf. Bei TVi = 400, 500, 600 und 700 ml führte der Albumin-Glutaraldehyd-Kleber zur kompletten Versieglung jeweils in 10, 10, 9 und 8 Lungen in der Versuchsgruppe, und 9, 7, 6 und 4 Lungen in der Kontrolgruppe. Der mittlere Bruchdruck war signifikant höher in Versuchsgruppe (41.0 ± 1.0 vs. 37.5 ± 4.2 cmH_2_O, *p* = 0.0195). Allerdings bestand kein Unterschied an Dehnung der versiegelten pleuralen Läsion in den beiden Gruppen (1.0 ± 0.4 vs. 1.5 ± 1.7 mm, *p* = 0.3772).

**Schlussfolgerung:**

Unsere in vitro Versuche zeigten, dass die rahmengestützte Applikationstechnik unkontrolliertes Abfließen des flüssigen Klebers effektiv verhindern und die Abdichtungswirksamkeit vom Albumin-Glutaraldehyd-Kleber verbessern kann. Wir empfehlen weitere Untersuchungen dieser Applikationstechnik in gut gestalteten, kontrollierten klinischen Studien.

## Background

Superficial parenchymal lung defects are common sequelae of lung surgery, particularly in patients with firm pleural adhesions or incomplete fissures. They result in alveolar air leaks (AAL), which are associated with prolonged chest tube duration and hospital stay as well as higher postoperative morbidity [1–3]. In the past decade surgical sealants have been increasingly used in treating AAL as adjuncts to conventional surgical closing techniques [4]. One of the most commonly used sealants is BioGlue™ (CryoLife Europa Ltd., Surrey, UK), which is composed of bovine serum albumin and glutaraldehyde [5]. Its clinical benefits for treating AAL have been proven in various clinical trials [6–8]. In addition, our previous in vitro experiment has confirmed the high sealing efficacy of BioGlue™, which is superior in resisting higher ventilation pressure [9].

BioGlue™ is delivered in liquid form, which makes it prone to unintentional run-off. Reported consequences include among others a blocked leaflet after mechanical aortic valve replacement resulting in a high transvalvular gradient [10]. Our personal experience in lung surgery confirms that BioGlue™ run-off after sealing superficial lung defects is almost inevitable and leads to hardened strands often far away from the original lesion site. These unintentional run-offs decrease the amount of sealant on the lesion which might reduce its sealing efficacy. Moreover, due to the rigid nature of hardened BioGlue™, overflowing sealant might impair expansion of adjacent lung parenchyma.

In the present study we used an established in vitro lung model to examine whether a special application technique based on a silicone frame that is placed around lung defects might improve the sealing efficacy of BioGlue [11].

## Methods

### Experimental protocol

Lungs of German landrace pigs were freshly excised in a local slaughterhouse. Within two hours following harvest, the lungs were dissected along the trachea until the tracheal bifurcation was reached. The caudal lobe was selectively intubated, ventilated and immersed in water to ensure impermeability. After connection to the ventilation machine (Evita, Dräger, Lübeck, Germany), the caudal lobe was ventilated in volume-controlled mode with a PEEP of 5 cmH_2_O, an I:E ratio of 1:2 and a frequency of 12/min. The caudal lobe was fully inflated when inspiratory tidal volume (TVi) ≥ 400 mL. Over-inflation of the lobe was observed with TVi ≥ 800 mL. A superficial parenchymal lesion was created in a previously marked area of 40 × 25 mm on the inflated caudal lobe with gentle pressure from a small drill with a roughened conic head, working from the margins towards the lesions center. Marker spots were then applied to the cranial and caudal edge of the lesion. Starting ventilation at TVi = 300 ml, TVi was increased by 100 ml in steps until a maximal inspiratory pressure (Pmax) of 40 cmH_2_O was reached. Following each increase in TVi, the expiratory tidal volume (TVe), resistance, compliance, as well as Pmax, mean inspiratory pressure (Pmean) and plateau inspiratory pressure (Pplat) were recorded after five cycles. AAL was calculated as the difference between TVi and TVe.

**Fig. 1:**
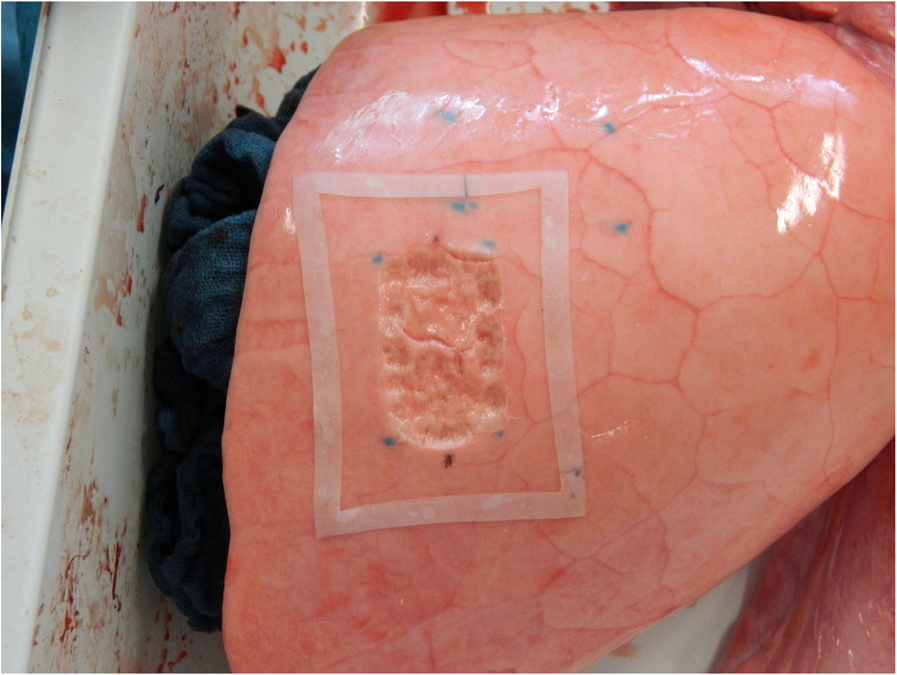
Placement of a silicone frame around a parenchymal lesion (40 × 25 mm)

**Fig. 2:**
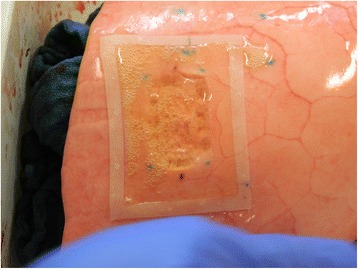
Applied BioGlue™ within the borders of the silicone frame

Lung lobes were randomly selected and subjected to either a standard technique sealing in accordance with CryoLife guidelines (control group, *n* = 10) or a modified technique relying on placement of a square silicone frame around the lesion site (study group, *n* = 10). Standard sealant application consists in carefully meandering along the lesion surface and, in accordance with user guidelines, respecting a safety margin of 1 cm to all sides. For our modified technique, a silicone frame (transparent silicone 60 ± 5 Shore, Erik’s NordOst GmbH, Hannover, Germany) customized to allow for the mentioned safety margin, was placed around the lesion on the inflated lower lobe (Fig. 1). Afterwards glue was applied within frame borders in the same meandering fashion. In both groups a period of 60 s was awaited until the glue hardened and full sealant adhesion was achieved (Fig. 2). In this experiment the sealant was applied exclusively in 2 ml syringes, using only one sample for each lesion.

The caudal lobe was then ventilated again with TVi rising slowly from 100 ml. Commencing at TVi = 400 ml the same parameters as prior were recorded, continuously screening for bubbles under water application. The distance between marker spots was measured with each increase for the evaluation of elasticity. Air leak was assessed through air bubble observation by two independent investigators. Any disagreement would be arbitrated by a third investigator. Sealing was considered successful, when no bubble was visible under submersion after five cycles of ventilation. This corresponds to grade 0 on the Macchiarini scale [11]. Sealing failure was determined once air bubbles were observed (grade 1 or higher). In the moment of sealant failure Pmax was recorded as burst pressure. Sealant failure was furthermore categorized into adhesive or cohesive failure. Adhesive failure was considered if the sealant failure occurred at the interface between sealant and parenchymal defect. Cohesive failure was defined as failure within the sealant. When cohesive or adhesive failure was observed before starting the test at TVi = 400 ml, this was considered application error.

### Statistical analysis

The normality of variables was tested using the Kolmogorov-Smirnov one-sample-test. Descriptive statistics are presented as mean ± standard deviation in case of normal distribution. Multiple linear regression was used to determine the ventilation parameters’ correlation with AAL. Statistical significance was assumed if *p* < 0.05. All statistical analysis was performed using SPSS (version 16.0 for Windows; SPSS Inc., Chicago, Illinois, USA).

## Results

Following a set of four pilot tests for our modified technique, a total of 20 consecutive tests were undertaken by means of the standard and frame application techniques in a randomized manner (see the Additional file 1).

**Fig. 3:**
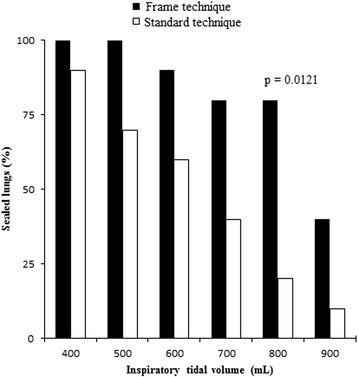
Sealing rates of BioGlue™ in study and control group

AAL prior to glue application were comparable in both groups (Table 1). Application error occured once during standard application. At TVi = 400, 500, 600, 700 and 800 ml, BioGlue™ achieved sealing in 10, 10, 9, 8 and 8 lobes in the study group, while 9, 7, 6, 4 and 2 lobes were sealed in the control group, respectively. Even in over-inflated lobes (TVi = 900 ml), superficial defects were still sealed in four tests in our study group, while only one lobe remained sealed in the control group. Sealing rates of both groups are presented in Fig. 3. Difference in sealing rate between both groups reached statistical significance at TVi = 800 ml (80 % vs. 20 %, *p* = 0.0121).

**Table 1:** Air leak assessment before sealant application

TVi (ml)	Study group (*n* = 10)	Control group (*n* = 10)	*p* value
400	73.0 ± 41.0	75.0 ± 36.9	0.9146
500	110.0 ± 47.7	116.0 ± 56.8	0.8110
600	167.0 ± 64.5	168.0 ± 71.0	0.9754
700	226.0 ± 69.0	225.0 ± 89.2	0.9791

*TVi* inspired tidal volume

Mean burst pressure was significantly higher in the study group than in the control group (41.0 ± 1.0 cmH_2_O versus 37.5 ± 4.2 cmH_2_O*, p* = 0.0195). Both groups exhibited only cohesive sealant failures. Concerning elasticity, there was no difference in expansion of the covered defect between both application techniques (1.0 ± 0.4 vs. 1.5 ± 1.7 mm, *p* = 0.3772).

## Discussion

As a highly effective sealant, BioGlue™ is regularly implemented in cardiovascular surgery for hemostasis [5]. In lung surgery it has gained widespread acceptance as an adjunct in treating AAL in recent years [7]. A major drawback of its application in liquid form on the surface of inflated lung tissue is sealant run-off, which in turn may impair sealing efficacy and trap surrounding parenchyma. Recently our group has developed a special application technique which basically consists in placing a silicone frame around the lesion site to prevent sealant run-off (frame technique). The present in vitro experiment was aimed to examine whether this special applicaiton technique might improve the sealing efficacy of BioGlue™.

To assess sealing efficacy we used an established in vitro lung model in the present study, which has been proven reliable in the previous experiments [9, 12]. In a randomized order BioGlue™ was applied onto standardized superfical lung defects by means of the standard technique according to the usage guide or the frame technique. The testing results of the standard application technique demonstrated a high sealing efficacy of BioGlue™ in treating AAL. The mean burst pressure was very close to the upper limit of the commonly applied ventilation pressure (40 cmH_2_O). However, when a silicone frame was placed around lesion site, run-off of the liquid sealant could be prevented and the sealing efficacy presented as sealing rate and burst pressure was significantly improved. In majour lung resection, division of incomplete fissures is often inevitable and causes superficial parenchymal lung defects and postoperative prolonged air leaks despite meticulous surgical technique. In many cases, the defect is not horizontal, rendering adaequate applicaion of BioGlue™ difficult. According to our results, it is reasonable to speculate that the frame-assisted application technique might facilitate sealing air leaks in this specific setting. Nevertheless, the potential clinical benefits and practical implications of this special application technique require confirmation from well-designed randomized clinical trials.

In the present in vitro experiment we used a square silicone frame to test the special application technique. The measurement of the lesion’s length before and after sealant application indicated that the silicone frame did not alter the elasticity of the underlying lung tissue. Despite the wide use of silicone in surgical practice, caution should be taken for potential side effects of this non-absorbable material. While concern has been arised about the chronic foreign body reactions and potential risk for carcinogenesis associated with silicone implants [13, 14], there have been report that implanted silicone may even be a protective factor concerning the development of carcinoma [15]. Taken together, the potential side effects of the present application technique deserves further investigation.

As video-assisted thoracic surgery (VATS) has been widely adopted and practiced in lung surgery in the last two decades, air leak sealing by means of topical sealant application through trocars has becomen a feasible approach [16]. A ample body of evidence demonstrates that prolonged air leaks are still one of the major complications after VATS major lung resections and limit the clinical benefits of this minimally invasive approach [17]. As liquid sealant BioGlue™ can also be applied thoracoscopically using a delivery tip extention. In this aspect, the present study might stimulate further investigation and improve the air leak management during VATS procedures.

One of the limitations of the present experiment is the certain inevitable variation in the size of tested procine lobes. To minimize this confounding feature, the lungs were harvested from the pigs in almost the same weight. As all lobes were fully inflated with a TVi of 400 ml, no significant differences were noted in this regard. In addition, the randomization of the application techniques might also contribute in reducing this bias. The authors recognize that the sealant applications were not blinded for the assessment of air tightness and the measurement of the lesion’s length in the present experiment. I t may have resulted in information bias, which was certainly minimized by randomization of the application techniques. Finally, the observation bias might have arisen due to the inevitable subjectiveness in the judgment of air bubbles, even though it was performed by two investigators independently. Nevertheless, the statistic analysis revealed significant results in favor to the application technique with silicone frame. We believe that our investigation is a further step to improve the application of fluid glue and useful to prevent prolonged postoperative air leaks after lung resection. Future efforts will need to be directed both towards assessing the effectiveness of the frame-assisted application technique in well designed clinical trails and towards examination of absorbable materials as frame.

## Conclusion

Our in vitro tests indicated that application by means of the frame technique prevents glue run-off and improve the sealing efficacy of BioGlue™. The implications of this special application technique should be further analyzed in well-designed, controlled clinical trials.

## Additional file

Additional file 1: Results of individual tests. (XLS 63 kb)

## Abbreviations

AAL: Alveolar air leak
PEEP: Positive end-expiratory pressure
Pmax: Maximal inspiratory pressure
Pmean: Mean inspiratory pressure
Pplat: Plateau inspiratory pressure
TVe: Expiratory tidal volume
TVi: Inspired tidal volume
